# Correction to “A cross‐sectional study of factors associated with nurses’ postoperative pain management practices for older patients”[Youngcharoen, P., & Aree‐Ue, S. (2023). A cross‐sectional study of factors associated with nurses' postoperative pain management practices for older patients. Nursing Open, 10(1), 90–98. https://doi.org/10.1002/nop2.1281].

**DOI:** 10.1002/nop2.2220

**Published:** 2024-06-28

The possible score of the CSACD is incorrect.

The CSACD consisted of 9 items with a 7‐point Likert scale from 1 to 7. Thus, the possible score ranges from 9 to 63 **not** 7 to 63 as well as a possible score of nurses' collaboration with physicians in Table 2 is 9–63.

We apologize for this error.

